# Horizontally Acquired Polysaccharide-Synthetic Gene Cluster From *Weissella cibaria* Boosts the Probiotic Property of *Lactiplantibacillus plantarum*

**DOI:** 10.3389/fmicb.2021.692957

**Published:** 2021-06-21

**Authors:** Yuqi Gao, Mingze Niu, Xiaohui Yu, Tingting Bao, Zhaowei Wu, Xin Zhao

**Affiliations:** ^1^College of Animal Science and Technology, Northwest A&F University, Yangling District, China; ^2^School of Physical Science and Technology, ShanghaiTech University, Shanghai, China; ^3^Department of Animal Science, McGill University, Montreal, QC, Canada

**Keywords:** *Lactiplantibacillus plantarum*, *cpsWc*, exopolysaccharides, biofilm, adhesion

## Abstract

*Lactiplantibacillus plantarum* are probiotic bacteria, maintaining the integrity of the gastrointestinal epithelial barrier, and preventing the infection of pathogenic bacteria. Exopolysaccharides (EPSs) are often involved in the probiotic property of *L. plantarum*. Here, we identified a new EPS-synthetic gene cluster, *cpsWc*, carrying 13 genes, laid on a large plasmid in a well-characterized probiotic *L. plantarum* strain LTC-113. The *cpsWc* gene cluster was horizontally acquired from *Weissella cibaria*, enhancing the biofilm formation ability of the host strain and its tolerance to harsh environmental stresses, including heat, acid, and bile. Transfer of *cpsWc* also conferred the probiotic properties to other *L. plantarum* strains. Moreover, *cpsWc* strengthened the adhesion of LTC-113 to intestinal epithelial cells. Both the *cpsWc*-carrying LTC-113 and its EPSs *per se* effectively attenuated the LPS-induced pro-inflammatory effect of intestinal epithelial cells, and inhibited the adhesion of pathogenic bacteria, such as *S. typhimurium* and *E. coli* by exclusion and competition. The newly identified *cpsWc* gene cluster emphasized the contribution of mobile EPS-synthetic element on the probiotic activity of *L. plantarum*, and shed a light on the engineering of probiotic bacteria.

## Introduction

*Lactiplantibacillus plantarum* is widely distributed environmental bacteria and is often considered a probiotic agent contributing to human health and food production (van Baarlen et al., 2009; Jiang and Yang, 2018). *L. plantarum* is capable of interfering with the adhesion and proliferation of pathogenic bacteria (Alizadeh Behbahani et al., 2019; Liu et al., 2019). *L. plantarum* also possesses immunomodulatory activity to coordinate the expression of cytokines in host immune cells (Zhao et al., 2018; Kim et al., 2020), and attenuates the inflammatory bowel disease and diarrhea (de Vrese and Marteau, 2007; van Baarlen et al., 2009; Le and Yang, 2018). In the food industry, *L. plantarum* strains are widely used to improve food quality and prevent certain bacterial and fungal spoilage (Papadopoulou et al., 2018; Xiao et al., 2020).

Most *L. plantarum* strains secrete one or more exopolysaccharides (EPSs), which are high-molecular-weight carbohydrate polymers, protecting the host strain from harsh environmental stresses and significantly contributing to the probiotic properties (Jiang and Yang, 2018). The biosynthesis of EPS in *L. plantarum* predominantly adopts the Wzx/Wzy-dependent pathway, in brief, which is comprised of five major steps, (i) Transportation and phosphorylation of monosaccharides and disaccharides, such as glucose, galactose, and rhamnose, by phosphotransferase system (PTS) or permease-assisted system; (ii) Formation of sugar nucleotides, such as UDP-glucose, UDP-galactose, and dTDP-rhamnose; (iii) Synthesis of repeating sugar units on a undecaprenol diphosphate anchor (C55) located in the inner membrane by various glycosyltransferases; (iv) Translocation of the repeating units from intracellular to the outer membrane by the Wzx flippase; (v) Polymerization of repeating units by the Wzy polymerase to form a long chain of EPS (Jiang and Yang, 2018; Zhou et al., 2019). Eventually, EPS is either released to the extracellular space or transferred to cell-wall peptidoglycan by LCP-family ligases to assist in forming the bacterial capsule (Chan et al., 2014; Li et al., 2020b). EPSs from *L. plantarum* strains vary in their bioactivities, according to the highly different monosaccharide composition, linkage position, branching pattern, and post-synthesis chemical modifications (Jiang and Yang, 2018; Zhou et al., 2019).

To date, four major EPS-synthetic gene clusters have been identified in *L. plantarum* strains, which are designated as *cps1*, *cps2*, *cps3*, and *cps4*. In the well-characterized *L. plantarum* WCFS1 strain, four *cps* gene clusters are all present in the bacterial genome and located in two separated regions that one contains the *cps1*, *cps2*, and *cps3* clusters in tandem, while another holds the *cps4* cluster (Remus et al., 2012). The *cps1* gene cluster controlled the molecular mass and the rhamnose composition of EPS (Remus et al., 2012). The *cps2* gene cluster managed the galactose composition of the EPS. Individual deletions of the *cps3* and *cps4* gene clusters showed no significant change in the monosaccharide composition of the EPS. Simultaneous deletion of the *cps2*, *cps3*, and *cps4* gene clusters significantly reduced the total EPS yield. However, the collaboration and communication of *cps* gene clusters were unclear. Moreover, other *L. plantarum* strains may possess differential *cps* gene composition and cluster configuration, leading to the diverse bioactivities of EPSs from *L. plantarum* strains (Jiang and Yang, 2018; Zhou et al., 2019). Furthermore, some EPS-synthetic gene clusters laid on mobile genetic elements, such as plasmid and transposon, conferring new EPSs with diverse structure, and function to donor strains (Jiang and Yang, 2018).

We have previously isolated a probiotic *L. plantarum* strain LTC-113 from the intestine of a healthy Tibet local chicken (Wang et al., 2018). LTC-113 can protect newly hatched chicks from intestinal barrier disruption by *Salmonella typhimurium* infection via regulating the expression of tight junction genes and inflammatory meditators and decreasing the *S. typhimurium* colonization (Wang et al., 2018). Moreover, another study also found LTC-113 significantly decreased chickens’ mortality and improved the health status of chickens breeding in high altitude regions (Wang et al., 2019). In this study, we sought to investigate whether EPS plays a role in the probiotic properties of LTC-113, and lay the groundwork for understanding the molecular basis of its probiotic activity using genomic, biochemical, biological, and engineering approaches.

## Materials and Methods

### Bacterial Strains

The probiotic *L. plantarum* strain LTC-113 was previously isolated from the intestine of a healthy Tibet local chicken (Wang et al., 2018), maintained in the DeMan, Rogosa, and Sharpe (MRS) broth at 37°C without shaking. The pathogenic *S. typhimurium* strain CVCC542 carrying spontaneous novobiocin resistance was used in this study (Wang et al., 2018), maintained in the Luria-Bertani (LB) broth at 37°C with constant shaking. The pathogenic *E. coli* strain O78 was used in this study, maintained in the Luria-Bertani (LB) broth at 37°C with constant shaking.

### Whole-Genome Sequencing and Comparative Genomics

Genomic DNA of *L. plantarum* LTC-113 was extracted using the EasyPure genomic DNA kit (TransGen, Beijing, China). The integrity of the extracted genomic DNA was checked by agarose gel electrophoresis and quantified by NanoDrop 2000. Next, the genomic DNA was disrupted into short fragments of approximately 350 bp, tailed with A end, ligated with adaptors, and PCR amplified to construct the sequencing library. Sequencing was performed on the Illumina HiSeq 2500-PE125 platform with MPS (massively parallel sequencing) Illumina technology at the Beijing Novogene Bioinformatics Technology Co., Ltd., Illumina PCR adapter reads and low-quality reads were filtered, and paired reads in good quality were assembled using the SOAPdenovo (version 2.21) into scaffolds. The scaffolds’ order was determined by alignment to a reference genome of *L. plantarum* strain WCFS1 (GenBank accession no. NC_004567) using SOAPaligner (version 2.21). Then the gaps between scaffolds were closed by PCR and sanger sequencing, therefore, resulting in a complete circular chromosome of *L. plantarum* LTC-113, and an episomal plasmid, termed pYZ1 (sequence information can be found in Supplementary Table 1). The *cps* gene clusters in *L. plantarum* LTC-113 were identified and annotated based on the results from Blastn searching against the genomes of *L. plantarum* WCFS1, LZ 227 (GenBank accession no. CP015857) and *W. cibaria* MG1 (GenBank accession no. JWHU01000000), as well as plasmids of Lp16H (GenBank accession no. CP006041) and C410L1 P1 (GenBank accession no. CP017955). The multiple sequence alignment results were visualized by using Easyfig.

### Plasmids and Oligonucleotides

Two complementing plasmids, p*cpsWcA-M* and p*cpsWcA-K*, were constructed by Gibson assembly based on a parent plasmid carrying a non-temperature-sensitive *Enterococcus* replication origin. Detailed plasmid sequences and primers used in plasmid construction can be found in Supplementary Table 1. To introduce these plasmids into *L. plantarum* strains, 1-mL overnight culture of *L. plantarum* was inoculated into 100-mL MRS broth, and incubated at 37°C until an OD_600_ of 0.5 was reached. The cells were pelleted by centrifugation at 10,000 × *g* and 4°C for 5 min. The pellet was washed three times with ice-cold 0.5 M sucrose. The pellet was finally resuspended to 1-mL 0.5 M sucrose and kept on ice until electroporation. Plasmid DNA (approximate 1 μg) was mixed with 40 μL cell suspension in an ice-cold electroporation cuvette (0.2 cm gap) and electroporated at 2.5 kV, 200 Ω, and 25 μF (Spath et al., 2012). After the pulse, 1-mL MRS broth was added to the cells and incubated at 37°C for 2 h. The bacteria were then plated on MRS plates supplemented with 5 mg/L chloramphenicol and incubated at 37°C for 2 days. The copy number of pYZ1 was determined by qPCR. A single copy housekeeping gene *gyrB* was selected as an internal reference. The copy number was calculated by the algorithm of 2^–ΔΔ*C*^*^*t*^* (ΔCt = Ct_*p*__*Y*__*Z*__1_−Ct*_*gyrB*_*). Each experiment was performed in triplicate. Primers used in qPCR were showed in Supplementary Table 1.

### Probiotic Properties of *L. plantarum* LTC-113 and Its Derivative

#### Biofilm

The biofilm formation ability of *L. plantarum* LTC-113 and its derivative strains were assayed by applying the microplate assay (Terraf et al., 2012). Briefly, 2 μL of each strain (approximate 2 × 10^5^ CFU) was inoculated in 96-well polystyrene microplates with 200 μL MRS, and incubated in at 37°C for 12 h. Unattached bacteria was washed away with 3-time PBS wash. The remaining attached bacteria was fixed by incubating at 60°C for 1 h, and then stained with 150 μL crystal violet for 15 min. Excess stain was rinsed with distilled water. The remaining dye was dissolved with 200 μL 95% ethanol and quantified by a microplate reader at 595 nm.

#### Aggregation

The overnight culture of *L. plantarum* LTC-113 and its derivative strains were harvested by centrifugation at 4,000 × *g* and 4°C for 10 min. The pellet was resuspended in PBS and adjusted to approximate 10^9^ CFU/mL. Next, the suspensions were incubated at 37°C. The optical density at 600 nm of upper layer suspension was measured at 4 and 16 h by a spectrophotometer without any distributing of the suspension. The aggregation ability was calculated as follows: Aggregation (%) = (1-ABS_*time*_/ABS_*initial*_) × 100%, where ABS_*time*_ is the OD_600_ measured at 4 or 16 h, and ABS_*initial*_ is the OD_600_ determined at 0 h (Han et al., 2017).

#### Growth Curve

To determine the growth curves of *L. plantarum* LTC-113 and its derivative strains. The 1-mL overnight culture of these strains were inoculated 1 L fresh MRS medium (in a 1-L airtight sterile bottle) and incubated at 37°C for 16 h. The optical density at 600 nm of the culture was measured at every 2 h by a spectrophotometer to record the growth curves.

#### Determination of EPS Concentration

The concentration of EPS in the culture supernatant was assay using a phenol-sulfate method. Briefly, the phenol-sulfate reagent was prepared by mixing 10 mL of 5% phenol and 50 mL 98% sulfate acid. To determine the concentration of EPS, 30 μL of culture supernatant was mixed with 90 μL of phenol-sulfate reagent, and incubated at 95°C for 20 min. The OD_490_ value was recorded and the concentration of EPS was calculated according to a pre-established standard curve using glucose gradients.

#### Purification of EPS

To purify the supernatant EPS from the culture of LTC-113 and its derivatives, bacteria was pelleted by centrifugation at 12,000 × *g* and 4°C for 10 min. Trichloroacetic acid was added to the supernatant to a final concentration of 20%, and incubated at 4°C for overnight. Precipitated proteins were removed by centrifugation at 12,000 × *g* and 4°C for 60 min. Then 2 volumes of absolute ethanol was added to the supernatant, and incubated at 4°C for 2 h. EPS was pelleted by centrifugation at 12,000 × *g* and 4°C for 10 min. The EPS was air dried and dissolved in H_2_O. Then, the EPS was subjected to ion-exchange chromatography using a HiTrap DEAE column (GE healthcare) with linear gradient elution. Fractions containing EPS were merged and concentrated by ultrafiltration, and subsequently subjected to size-exclusion chromatography using a HiLoad 16/600 Superdex 200 pg column (GE healthcare).

#### Tolerance to Environmental Stresses

To determine the thermal tolerance of *L. plantarum* LTC-113 and its derivative strains, these bacteria were inoculated in MRS broth and incubated at 37°C and 43°C, respectively, and the final OD_600_ was recorded. To determine the tolerance to low pH and bile salt, the overnight culture of *L. plantarum* LTC-113 and its derivative strains were harvested by centrifugation at 4,000 × *g* and 4°C for 10 min (Li et al., 2017). The pellet was washed in PBS buffer, and resuspended in MRS pre-adjusted to 2.0 and 3.0, and MRS supplemented bile salt at 0.3 and 1%, respectively, and incubated at 37°C for 2 h. The suspensions were then pelleted by centrifugation at 4,000 × *g* and 4°C for 10 min, and washed with neutral PBS buffer for twice. Subsequently, ten-fold serial dilution of the cells were prepared and 100 μL of each dilution was plated onto MRS plate, and incubated at 37°C for 24 h. The percentage of the viable bacteria was calculated.

#### Adhesion Assays

The adhesions of *L. plantarum* LTC-113 and its derivative strains to human colon adenocarcinoma cell line Caco-2 were determined as previously described with minor modifications (Pisano et al., 2014). Briefly, Caco-2 cells were routinely maintained in DMEM medium supplemented 10% fetal bovine serum at 37°C in 5% CO_2_. Caco-2 cells were cultured to 70–80% confluence in 500 μL of medium in 24-well plates for the adhesion assays. Then, 100 μL of PBS-based *L. plantarum* suspension (approximate 2 × 10^8^ CFU/mL) was added into the Caco-2 cells at an MOI of 1:100. The mixture was continued incubating for 2 h. The monolayer was washed five times with PBS, and trypsinized to detach the cells and bacteria. Subsequently, ten-fold serial dilution of the bacteria was prepared and 100 μL of each dilution was plated onto MRS plate, and incubated at 37°C for 24 h. The percentage of the viable *L. plantarum* was calculated. To test the adhesion of enzymatic-shaved bacteria, LTC-113 and its derivatives were treated with 0.25% trypsin for 60 min at 37°C. The bacteria were washed with PBS for three times, and then subjected to the adhesion assay.

### Anti-inflammatory Assays

To induce the pro-inflammatory effect, Caco-2 cells were maintained in serum-free DMEM for overnight and then treated with 10 μg/mL of LPS for 24 h in the presence or absence of EPS (100 μg/mL) or bacteria (approximate 2 × 10^7^ CFU). The expression level of IL-8 was determined by qRT-PCR. Briefly, total RNA was extracted from the cells using TRIzol reagent (Invitrogen). RNA was then reverse-transcribed into cDNA using the PrimeScript RT Reagent Kit with gDNA Eraser (Takara). Subsequently, qRT-PCR was performed using TransStart Green qPCR SuperMix (Transgen). The mRNA level of IL-8 was normalized to GADPH as an internal reference. Primers used in qRT-PCR are listed in Supplementary Table 1.

### Pathogen Exclusion, Competition, and Displacement Assay

For pathogen exclusion assay, cells were first incubated with *L. plantarum* at an MOI of 1:100 for 2 h and washed with PBS for twice prior to infection of *S. typhimurium* at an MOI of 1:100 for additional 2 h (Wang et al., 2017). After incubation, the cell surface was washed five times with PBS, and trypsinized to detach the cells and bacteria. Subsequently, ten-fold serial dilution of the bacteria was prepared and 100 μL of each dilution was plated for CFU counting. The percentage of the viable *S. typhimurium* was calculated. For competition assay, *S. typhimurium* and *L. plantarum* were added to Caco-2 cells simultaneously. For displacement assay, *S. typhimurium* was first added to Caco-2 cells and incubated for 2 h prior to supplementing the *L. plantarum* strains. In parallel, the pathogenic inhibition assay was also performed using EPS only (100 μg/mL) and enzymatic-shaved bacteria. For CFU counting, LB agar supplemented with novobiocin was used to selectively isolate *S. typhimurium*, and MacConkey agar was used for *E. coli*.

### Statistical Analysis

All experiments were performed in triplicate. Microsoft Windows Excel 2019 and GraphPad Prism 8.2.1 were used in data analysis. Significant level in difference was determined by one-way ANOVA analysis with Tukey *post*-test at *p* < 0.05.

## Results

### Comparative Genomics of the EPS-Synthetic Gene Clusters in *L. plantarum* LTC-113

**FIGURE 1:**
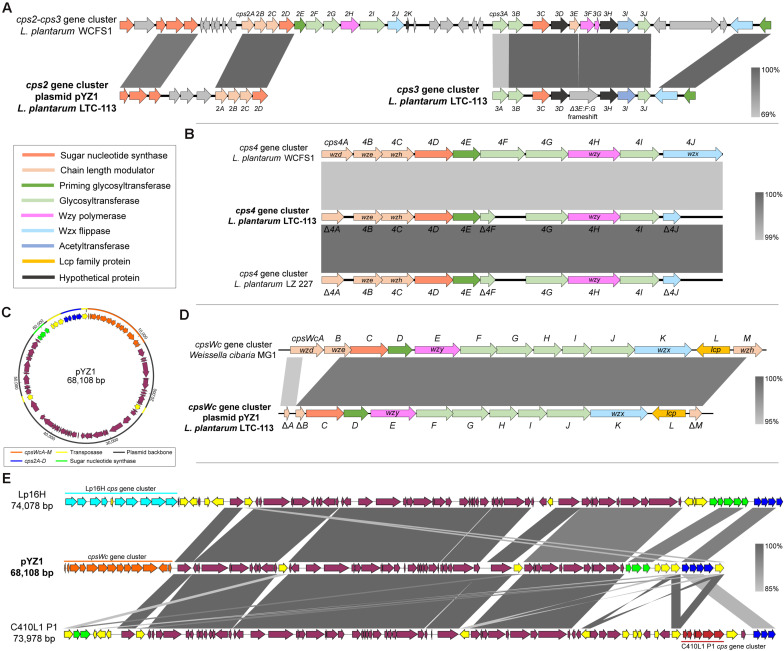
Comparative genomics of cps gene cluster in *L. plantarum* LTC-113. **(A)** Schematic representation of the *cps2* and *cps3* gene cluster of *L. plantarum* LTC-113 compared to these in *L. plantarum* WCFS1. Genes were showed as arrows in different colors. **(B)** comparative analyses of the *cps4* gene cluster of LTC-113 compared with that in WCFS1 and LZ 227. **(C)** The circular map of the plasmid pYZ1. **(D)** Sequence alignment between the *cpsWc* gene cluster on pYZ1 and that on *W. cibaria* MG1. **(E)** Multiple sequence alignment of plasmids, pYZ1, Lp16H, and C410L1 P1.

Whole-genome sequencing of *L. plantarum* LTC-113 was carried out to systematically investigate the molecular basis of EPS synthesis. The complete genome of LTC-113 contains a single circular chromosome of 3.14 Mb with a large plasmid of 68.1 Kb, designated as pYZ1 in this study. Four *cps* gene clusters, *cps2*, *cps3*, *cps4*, and *cpsWc*, responsible for EPS biosynthesis, were identified in LTC-113. Two of them (*cps3* and *cps4*) were located on the chromosome, and another two (*cps2* and *cpsWc*) were present in the plasmid pYZ1 (Figures 1A–D). In the best characterized *L. plantarum* model strain WCFS1, *cps1*, *cps2*, and *cps3* are clustered together in a genomic region of 51 Kb. In contrast, *cps1* gene cluster was absent in LTC-113. The *cps2* gene cluster on the plasmid pYZ1 was imperfect, which only retains four-function genes, *cps2ABCD* (Figures 1A,C). Cps2ABC are tyrosine protein kinases (also known as Wzd, Wze, and Wzh), which regulate the Wzy polymerase and modulate the chain length of EPS. The *cps2D* encodes a UDP-N-acetylglucosamine 4-epimerase, which manages the interconversion between UDP-N-acetyl-D-glucosamine and UDP-N-acetyl-D-galactosamine. The mobility of the truncated *cps2* gene cluster was possibly conferred by the surrounding transposases (Figure 1C). Similar configurations of the *cps2* gene cluster were also observed in other *L. plantarum* large plasmids (Figure 1E). The genomic *cps3* gene cluster of LTC-113 is similar to that of WCFS1 (Figure 1A). Cps3AB and Cps3J are predicted glycosyltransferases. Cps3C encodes a UDP-galactopyranose mutase, converting UDP-*D*-galactopyranose to UDP-*D*-galacto-1,4-furanose. Cps3D and Cps3H are hypothetical proteins. Cps3E shares similarity with tyrosine protein kinase, which may regulate the chain length of EPS. Cps3F and Cps3G are Wzy homologs. The Wzx flippase and the priming glycosyltransferases were separated at the downstream of the *cps3* gene cluster in WCFS1, whereas, they were clustered together in the *cps3* gene cluster of LTC-113. Interestingly, a 52-bp deletion and a 1-bp deletion were identified at the subterminal region of *cps3E* and the beginning region of *cps3F* in the *cps3* gene cluster of LTC-113, respectively, resulting in frameshifted fusion of *cps3E*, *cps3F*, and *cps3G*, consequently, inactivating the Wzy polymerase (Fig. 1A). The *cps4* gene cluster is conserved in the chromosomes of all *L. plantarum* strains. Cps4ABC are chain-length modulators as Cps2ABC. Cps4D is also a UDP-N-acetylglucosamine 4-epimerase. Cps4E is a priming glycosyltransferase, and Cps4F, Cps4G, and Cps4I are glycosyltransferases to form sugar repeat units. Cps4H and Cps4J are predicted Wzy polymerase and Wzx flippase, respectively. In LTC-113 strain, *cps4A*, *cps4F*, and *cps4J* are truncated by pre-mature translation termination (Figure 1B).

Intriguingly, a foreign *cps* gene cluster originated from another probiotic species, *Weissella cibaria*, which was identified in the plasmid pYZ1 of LTC-113, and designated as the *cpsWc* gene cluster in this study (Figure 1C and Supplementary Figure 1). The *cpsWc* gene cluster consisted of an 11-gene operon containing *cpsWcA-K* and two individual genes (Figure 1D). Compared with the original *cpsWc* gene cluster from *W. cibaria*, the chain-length modulator, *cpsWcA*, *cpsWcB*, and *cpsWcM* were truncated in the plasmid-borne *cpsWc* gene cluster (Figure 1D). While other components, including a UDP-glucose 4-epimerase (*cpsWcC*), a priming glycosyltransferase (*cpsWcD*) and other glycosyltransferases (*cpsWcFGHIJ*), a Wzy polymerase (*cpsWcE*), and a Wzx flippase (*cpsWcK*) are intact (Fig. 1D). Moreover, an LCP family protein (*cpsWcL*) has also been identified, which was predicted to catalyze the transfer of EPS from the undecaprenol diphosphate anchor to the cell-wall peptidoglycan, forming the bacterial capsule. As shown in Figure 1E, comparative genomics indicated that the plasmid pYZ1 shared a similar plasmid backbone with other large *L. plantarum* plasmids. The *cps* insertion locus is highly variable in these plasmids due to the frequent genetic interchange via transposase-mediated intraspecies or interspecies horizontal gene transfer. Furthermore, the maintaining stability of the plasmid pYZ1 was also determined. As shown in Supplementary Figure 2, the plasmid pYZ1 retained a relatively stable copy number of 1∼2 plasmids per one bacteria cell during a 5-time consecutive passage at 30°C and 37°C. However, elevating the temperature to 43°C significantly inhibited the replication of pYZ1 and reduced its copy number to about 0.04 plasmids per one bacteria cell after a 5-time consecutive passage.

Taken together, the previously characterized probiotic *L. plantarum* LTC-113 strain harbored four *cps* gene clusters. However, the *cps2*, *cps3*, and *cps4* gene clusters were incomplete or imperfect by carrying inactivated essential components, such as Wzy polymerase or Wzx flippase. The only remaining plasmid-borne *W. cibaria*-originated *cpsWc* gene cluster were predicted to be functional for EPS biosynthesis in the *L. plantarum* LTC-113 strain, leading to a hypothesis that the *cpsWc* gene cluster may impact the probiotic properties of the host strain.

### The Contribution of the *cpsWc* Gene Cluster to the Probiotic Activities of *L. plantarum* LTC-113

**FIGURE 2:**
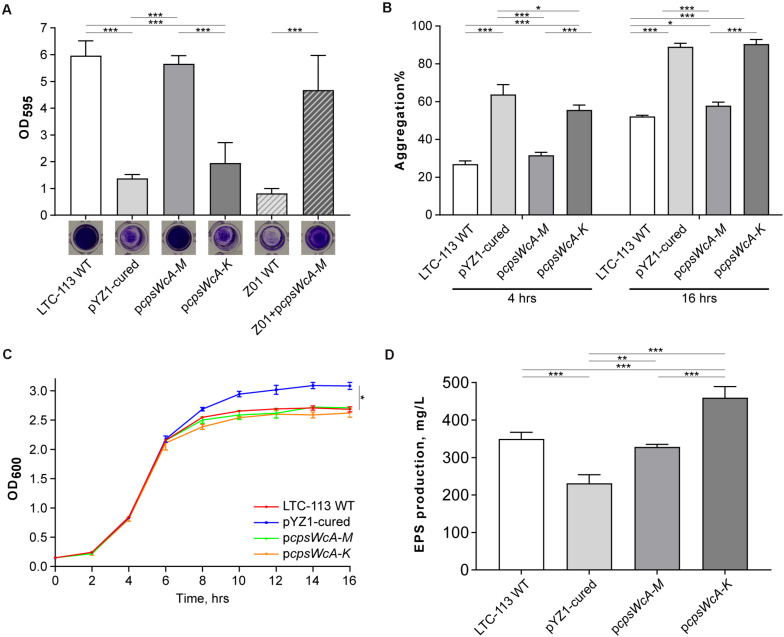
Biochemical characterizations of LTC-113 and its derivative strains. **(A)** The biofilm formation ability of LTC-113 and its derivative strains. **(B)** The bacterial aggregation of LTC-113 and its derivative strains at 4 and 16 h. **(C)** The growth curve of LTC-113 and its derivative strains during a 16-hour time course. **(D)** The concentration of supernatant EPS of LTC-113 and its derivative strains. Values represent mean ± SD of the mean; *P < 0.05; **P < 0.01; ***P < 0.001.

To verify whether the *cpsWc* gene cluster contributed to the probiotic properties of the *L. plantarum* LTC-113 strain, a pYZ1-cured derivative strain was first isolated via thermo-mediated plasmid curing (Supplementary Figure 2). We found that curing the pYZ1 plasmid significantly reduced the biofilm-formation ability of LTC-113 (Figure 2A). To rule out the effect from the plasmid backbone on biofilm formation, we complemented an artificial plasmid (p*cpsWcA-M*) carrying an 11.5-Kb region containing the complete *cpsWc* gene cluster (*cpsWcA-M*) into the pYZ1-cured strain. The complemented strain showed similar biofilm-formation ability as the LTC-113 wild-type strain, indicating the *cpsWc* gene cluster significantly contributed to the biofilm formation in LTC-113 (Figure 2A). Moreover, the LCP family protein was usually linked to the bacterial capsule and biofilm (Kawai et al., 2011; Chan et al., 2014). Therefore, another artificial plasmid (p*cpsWcA-K*) carrying a 9.8-Kb region containing the *cpsWcA-K* was constructed and introduced into the pYZ1-cured strain. Deletion of the *cpsWc*-specific LCP family protein (*cpsWcL*) also significantly reduced the ability to form biofilm in LTC-113 (Fig. 2A), indicating that the *cpsWc*-derived EPS may require a specific LCP family protein (*cpsWcL*) to mediate the transfer from undecaprenol diphosphate anchor to cell-wall peptidoglycan, eventually, facilitating the formation of biofilm. In addition, through transmission electron microscope (TEM), we found that the surface of LTC-113 was coarse, in contrast, curing pYZ1 smoothed the surface (Supplementary Figure 3). Complementation of the complete *cpsWc* gene cluster restore the surface phenotype, reinforcing the suggestion that the *cpsWc*-specific LCP family protein was likely dedicated to function on its cognate EPS molecules. Furthermore, we also tested whether the *cpsWc* gene cluster functions in other *L. plantarum* strains by introducing the p*cpsWcA-M* plasmid to a weak-biofilm-formation strain Z01. As shown in Figure 2A, the *cpsWc* gene cluster also significantly boosted the biofilm formation in the Z01 strain.

In this study, we found the pYZ1-cured LTC-113 strain showed significantly higher speed in aggregation than the wild-type strain and complementing the complete *cpsWc* gene cluster restored the aggregation speed (Figure 2B). However, deletion of the *cpsWc*-specific LCP family protein (*cpsWcL*) also significantly facilitated the aggregation. These data indicated that the majority of *cpsWc*-derived EPS may attach to the bacterial cell surface and reduced the density of the bacteria, consequently, slowing down the aggregation speed. Moreover, the growth curves of these strains showed that the pYZ1-cured strain yielded more bacteria load than the LTC-113 wild-type strain and the two *cpsWc* complement strains in stationary phase after 16 h of incubation, however, no difference was observed among them in the logarithm phase (Figure 2C), suggesting that the *cpsWc*-derived EPS biosynthesis majorly occurred in the stationary phase and consumed an amount of cell resources, therefore, reducing the final bacterial yield (Supplementary Figure 4). Furthermore, the supernatant EPS concentrations were also determined. LTC-113 produced approximately 350 mg/L EPS (Figure 2D). Curation of pYZ1 plasmid significantly reduced the supernatant EPS yield to about 230 mg/L, indicating that the imperfect genomic *cps3* and *cps4* gene cluster may mutually compensate each other to generate EPS (Figure 2D). We also found that EPSs from LTC-113 and the pYZ1-cured strain were heterologous with a broad range of molecular weight (Supplementary Figure 5), probably due to the deficiency in chain length modulator in *cps* gene clusters. The complement of the complete *cpsWc* gene cluster restored the supernatant EPS production to the same level as the LTC-113 wild-type strain (Figure 2D). Interestingly, deletion of the *cpsWc*-specific LCP family protein (*cpsWcL*) significantly elevated the EPS production to about two-fold of the pYZ1-cured strain, which suggested that the *cpsWc*-derived EPS was released to the culture supernatant. This finding reinforced the postulation that CpsWcL was responsible for transferring the EPS product to cell-wall peptidoglycan.

**FIGURE 3:**
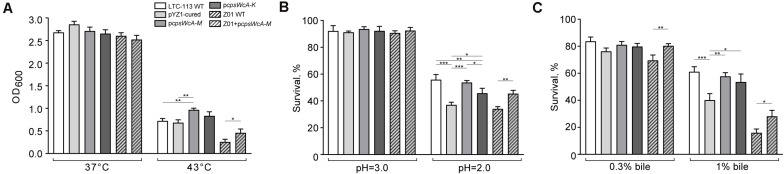
Tolerance of *L. plantarum* strains to environmental stresses. **(A)** The comparison of final bacterial density of LTC-113 and its derivative strains at 37°C and 43°C after 16-h incubation. **(B)** The survival rate of LTC-113 and its derivative strains after 2-h treatment at pH = 2.0 and 3.0, respectively. **(C)** The survival rate of LTC-113 and its derivative strains after 2-h treatment in 0.3 and 1% bile, respectively. Values represent mean ± SD of the mean; *P < 0.05; **P < 0.01; ***P < 0.001.

In addition, we also found that the *cpsWc* gene cluster was capable of elevating the tolerance of the host *L. plantarum* to environmental stresses, such as high temperature, low pH, and high concentration of bile. As shown in Figure 3A, incubation at 43°C substantially alleviated the growth of *L. plantarum*. Although the pYZ1-cured strain showed no difference in thermal tolerance with the wild-type strain, the p*cpsWcA-M* complementing strain exhibited significantly higher tolerance to high temperature since the backbone of the complementing plasmid was not temperature-sensitive. In contrast, the pYZ1 plasmid tended to lose during the incubation at a high temperature (Supplementary Figure 2). Moreover, the *cpsWc* gene cluster conferred the host strain higher tolerance to an extremely acidic environment (pH = 2.0) and high concentration of bile (1%) (Figures 3B,C). Furthermore, introducing the p*cpsWcA-M* plasmid into another *L. plantarum* strain Z01 also boosted its tolerance to these environmental stresses (Figures 3A–C).

Taken together, the *cpsWc* gene cluster was fully functional in EPS biosynthesis at the expense of consuming cell resources. The majority of *cpsWc*-derived EPS was possibly ligated to the cell-wall peptidoglycan, facilitating biofilm formation and alleviating the aggregation in stationary culture and elevating the tolerance of the host strain to harsh environmental stresses.

### The *cpsWc* Gene Cluster Enhanced the Compatibility of LTC-113 to Epithelial Cells

**FIGURE 4:**
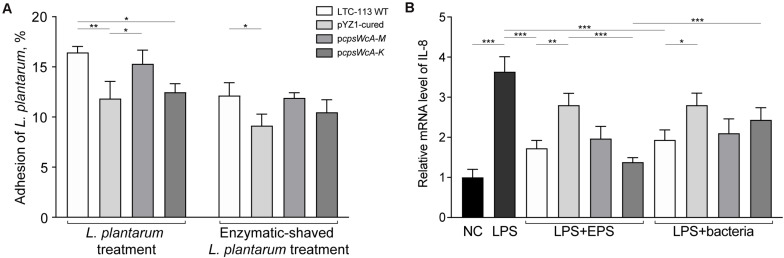
Adhesion of *L. plantarum* strains to intestine epithelial cells and its inflammation regulation ability. **(A)** The adhesion abilities of LTC-113 and its derivative strains to Caco-2 cells with or without trypsin shaving. **(B)** The anti-inflammatory effect by EPS only or bacteria. Values represent mean ± SD of the mean; *P < 0.05; **P < 0.01; ***P < 0.001.

Probiotic *L. plantarum* strains may protect the gastrointestinal tract by inhibiting pathogenic adhesion to the intestine epithelial cells. To investigate the contribution of the *cpsWc* gene cluster on inhibiting pathogenic adhesion, we first evaluated the adhesion of LTC-113 and its derivative strains to an intestine epithelial cell line (Caco-2 cells). As shown in Figure 4A, the adhesion of *L. plantarum* was significantly reduced by 28.0 and 24.1%, respectively, by curing the plasmid pYZ1 and complementing the p*cpsWcA-K* plasmid. While the complement of p*cpsWcA-M* showed no difference with the wild-type in epithelial cell adhesion. Similar results were also obtained using trypsin-treated LTC-113 and its derivatives. In addition, both LTC-113 and its EPS *per se* significantly countered the LPS-induced pro-inflammatory effect of intestinal epithelial cells (Figure 4B). Curation of pYZ1 erased the anti-inflammatory effect. Complementation of the complete *cpsWc* cluster partially restored the effect. Interestingly, deletion of LCP led to the release of more *cpsWc*-derived EPSs, thereby, significantly elevated the anti-inflammatory effect (Figure 4B).

### The *cpsWc* Gene Cluster Conferred LTC-113 Inhibitory Activity on Pathogenic Bacteria

**FIGURE 5:**
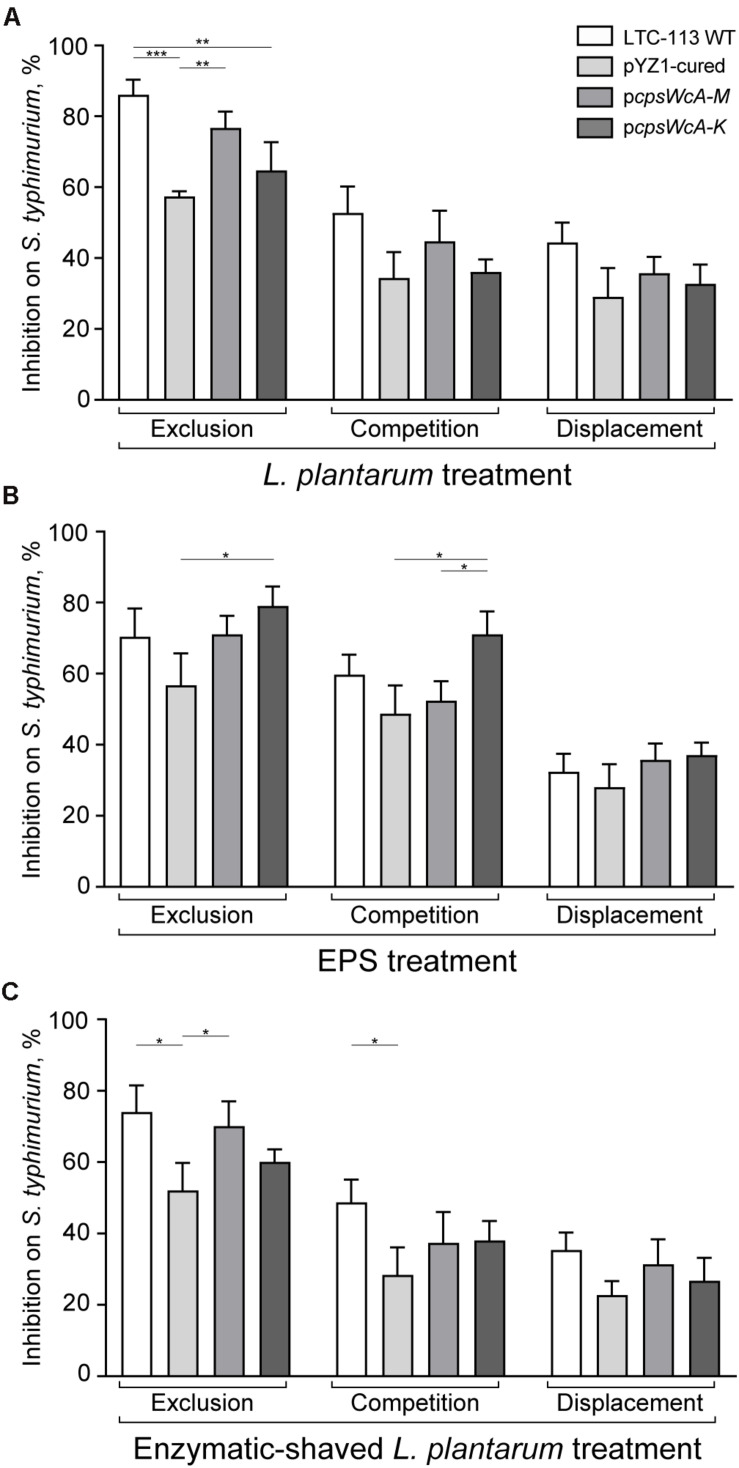
Inhibition on pathogenic *S. typhimurium* by *L. plantarum*. **(A)** The inhibitory activities of LTC-113 and its derivative strains on *S. typhimurium* in three experimental models, including exclusion, competition, and displacement. **(B)** Inhibition effect of EPS only. **(C)** Inhibition effect of trypsin-shaved bacteria. Values represent mean ± SD of the mean; *P < 0.05; **P < 0.01; ***P < 0.001.

The inhibitory activity of LTC-113 and its derivative strains on the adhesion of *S. typhimurium* to the intestine epithelial cells was assessed. To build an exclusion model, the Caco-2 cells were first incubated with LTC-113 and its derivative strains, respectively, prior to the infection of *S. typhimurium*. As shown in Figure 5A, the wild-type LTC-113 can efficiently inhibited the adhesion of *S. typhimurium* by 86.0%, whereas the inhibition was reduced to 57.3% by curing the pYZ1 plasmid. Complementing the complete *cpsWc* gene cluster restored the inhibition to the level of the wild-type strain. However, the complement of p*cpsWcA-K* was unable to fully reconstitute the inhibition ability. The results on inhibition were consistent with the findings in adhesion of *L. plantarum* alone to Caco-2 cells, suggesting that pre-treatment of *L. plantarum* protected the intestine epithelial cell from *S. typhimurium* infection. Subsequently, the inhibition on *S. typhimurium* was also tested in a competition model that simultaneously treating the cells with *S. typhimurium* and *L. plantarum*, and a displacement model that supplementing *L. plantarum* post the treatment of *S. typhimurium*. In the two models, the *cpsWc* gene cluster also contributed to the inhibitory activity of *L. plantarum* on the adhesion of *S. typhimurium* to intestine epithelial cells (Figure 5A). In parallel, treatment using EPS alone and enzymatic-shaved bacteria also generated similar results. In addition, the inhibition assay was also performed using pathogenic *E. coli*, and similar results were obtained (Supplementary Figure 6).

## Discussion

The major finding of this study was that a *W. cibaria*-originated *cpsWc* gene cluster was identified in a *L. plantarum* strain LTC-113, which was responsible for the probiotic activity of the host strain, facilitating the inhibition of *S. typhimurium* infection to intestine epithelial cells. Plasmid encoded *cps* gene clusters in *Lactiplantibacillus* was less reported, and the acquisition of foreign *cps* gene cluster through interspecies horizontal gene transfer was extremely rare (Jiang and Yang, 2018). The plasmid-borne *cpsWc* gene cluster showed the highest sequence similarity to that in *W. cibaria* strain MG1 (Lynch et al., 2015). *W. cibaria* was a well-known probiotic lactic acid bacteria species, and the MG1 strain was widely used in the food industry for yogurt, sourdough, and beverage fermentation (Zannini et al., 2013; Lynch et al., 2015; Wang et al., 2017). MG1 was known for its slime-producing property, i.e., secreting a homoexopolysaccharide (HoPS), dextran, in significant quantities (Zannini et al., 2013). However, the dextran production was predominantly linked to dextransucrase, which was not present in the *cpsWc* gene cluster (Lynch et al., 2015). Moreover, the *cpsWc* gene cluster was found to lay on a sequence contig of 21.1 Kb, which was the smallest contig present in the whole-genome shotgun sequencing of MG1, and in addition, MG1 was the only strain to harbor the *cpsWc* gene cluster in all sequence-characterized *W. cibaria* strains, leading to a postulation that the *cpsWc* gene cluster was a rare genetic element floating among *W. cibaria*, *L. plantarum*, and possibly other probiotic species, boosting the tolerance to harsh environmental stress in a plug-and-play style. In a recent study, three *eps* genes located on a *Levilactobacillus brevis* plasmid of 42.4 Kb, were found responsible for the EPS production, cell aggregation, and bile resistance (Fukao et al., 2019). Moreover, introducing the 3-gene set into other *L. plantarum* strains also resulted in similar phenotypic changes (Fukao et al., 2019). These findings were consistent with current study, collectively demonstrating the practicability of engineering new probiotic *L. plantarum* strains by introducing novel EPS-synthetic genes or clusters via stable plasmids or integrative genomic islands.

The LytR-CpsA-Psr (LCP) family proteins were widely identified in most Gram-positive bacteria species, exerting critical cell functions, including maintenance of cell shape and structural integrity, and multiple aspects of pathogenesis (Kawai et al., 2011; Chan et al., 2014). LCP enzymes were proposed to catalyze the transfer of undecaprenol-linked intermediates onto the C6-hydroxyl of MurNAc in peptidoglycan, thereby promoting the attachment of wall teichoic acid (WTA) and acidic polysaccharide to the cell wall (Kawai et al., 2011; Chan et al., 2014). In *Streptococcus pneumoniae* and *Streptococcus agalactiae*, the LCP-encoding gene was conserved at the beginning of the capsule biosynthetic operon, and its inactivation led to reduced capsule formation (Bentley et al., 2006; Yother, 2011). In our previous work, deletion of a genomic LCP gene in *Staphylococcus aureus* significantly reduced the adhesion ability to multiple epithelial cells (Li et al., 2020a). In this study, a novel *cpsWc*-specific LCP enzyme was characterized in the *cpsWc* gene cluster, considered to carry out the final step of EPS biosynthesis, transferring the *cpsWc*-derived EPS to the cell-wall peptidoglycan. The final step was critical for the manifestation of EPS-derived biological functions, as the EPS product tended to release to the culture supernatant if the LCP family protein was impaired. We found that the chromosome-encoded LCP enzymes may partially compensate for the activity of the *cpsWc*-specific LCP. Nevertheless, the *cpsWc*-specific LCP enzyme seemed dedicated to acting on the *cpsWc*-derived EPS.

Exopolysaccharides are known to enhance the bacterial tolerance to harsh environmental stress, maintaining the viability of the bacteria during industrial manufacturing and protecting it during delivery to the gastrointestinal tract (Dertli et al., 2015; Jiang and Yang, 2018; Fukao et al., 2019). EPSs also play a role in the inflammatory response of the host epithelial cells (Dertli et al., 2015; Jiang and Yang, 2018). Previously, LTC-113 showed excellent probiotic activity in protecting newly hatched chicken from *S. typhimurium* infection and decreasing the mortality (Wang et al., 2018, 2019). Data from current study revealed that the *cpsWc*-derived EPSs are the main driving power for the probiotic property. Similarly, Živković et al. (2016) showed that the EPS produced by *Lacticaseibacillus paracasei* subsp. *Paracasei* BGSJ2 efficiently prevented the Caco2-cells from the adhesion of *E. coli*. Another study also demonstrated that *Lactobacillus johnsonii* FI9785 administration inhibited the colonization of *Clostridium perfringens* and *E. coli* in twenty-day old chicks (Dertli et al., 2015). In contrast, other studies found that EPS reduced microorganisms’ binding ability by covering specific surface adhesins (Castro-Bravo et al., 2018; Jiang and Yang, 2018). The divergence in structural factors of EPS, including the monosaccharide composition, linkage position, branching pattern, and modifications, maybe the reason for the variable adhesion ability of bacteria.

Moreover, LTC-113 was demonstrated to regulate the expression of tight junction genes, such as claudins and occludin, and inflammatory mediators, including myeloperoxidase (MPO), LITAF, IL-1β, and IL-6, thereby maintaining the integrity of the intestinal barrier (Wang et al., 2018). In this study, we found that LTC-113 and its EPSs were capable of reducing the LPS-induced pro-inflammatory effect of intestinal epithelial cells. Similarly, Chen et al. (2019) demonstrated that the EPS administration from *Streptococcus thermophilus* MN-BM-A01 could counteract the disruption of intestine barriers by DSS in mice and prevent disease progression of colitis. Another study also found that the EPS from *L. plantarum* NCU116 prevented the intestinal barrier disruption by DSS in mice, and reduced the permeation of dextran-FITC into serum (Zhou et al., 2018).

In summary, the *W. cibaria*-originated *cpsWc* gene cluster conferred *L. plantarum* LTC-113 remarkable probiotic properties, i.e., enhanced biofilm formation, boosted tolerance to heat, acid, and bile stresses, elevated affinity to intestinal epithelial cells and anti-inflammatory effect, and promoted inhibitory effect on pathogenic bacteria. However, the composition and structure of the *cpsWc*-derived EPSs remain elusive. Consequently, further investigations are required to fully reveal its molecular basis and realize its engineering potential to create new functional probiotics.

## Data Availability Statement

The datasets presented in this study can be found in online repositories. The names of the repository/repositories and accession number(s) can be found in the manuscript/Supplementary Materials.

## Author Contributions

YG, ZW, and XZ conceived the initial study, prepared the figures, and wrote the manuscript. YG, MN, XY, and TB conducted the experiments. All authors reviewed and approved the manuscript, contributed to the article, and approved the submitted version.

## Conflict of Interest

The authors declare that the research was conducted in the absence of any commercial or financial relationships that could be construed as a potential conflict of interest.

## Supplementary Material

Supplementary Figure 1 The proposed EPS biosynthesis pathway for the cpsWc gene cluster.

Supplementary Figure 2 Copy number change of pYZ1 during serial passage at different temperatures. The wild-type LTC-113 strain was culture at 30°C, 37°C, and 43°C. The copy number of the plasmid pYZ1 was measured by qPCR.

Supplementary Figure 3 Transmission electron microscope analysis of LTC-113 and its derivatives.

Supplementary Figure 4 The supernatant EPS concentration curve in a 20-hrs time course.

Supplementary Figure 5 Analytical size-exclusion chromatography for EPS from LTC-113 and the pYZ1-cured strain.

Supplementary Figure 6 Adhesion inhibition of pathogenic *E. coli* by *L. plantarum*. The inhibitory activities of LTC-113 and its derivative strains on the adhesion of *E. coli* in three experimental models, including exclusion, competition, and displacement. Values represent mean ± SD of the mean; **P* < 0.05; ***P* < 0.01; ****P* < 0.001.

Supplementary Table 1 Genomes, plasmids, and oligonucleotides used in this study.

Supplementary Table 2 CFU counting data for Figure 3.

Supplementary Table 3 CFU counting data for Figure 4A.

Supplementary Table 4 CFU counting data for Figure 5.

## References

[B1] Alizadeh Behbahani B. Noshad M. Falah F. (2019). Inhibition of *Escherichia coli* adhesion to human intestinal Caco-2 cells by probiotic candidate *Lactobacillus plantarum* strain L15. *Microb. Pathog.* 136:103677. 10.1016/j.micpath.2019.103677 31437574

[B2] Bentley S. D. Aanensen D. M. Mavroidi A. Saunders D. Rabbinowitsch E. Collins M. (2006). Genetic analysis of the capsular biosynthetic locus from all 90 pneumococcal serotypes. *PLoS Genet.* 2:e31. 10.1371/journal.pgen.0020031 16532061 PMC1391919

[B3] Castro-Bravo N. Wells J. M. Margolles A. Ruas-Madiedo P. (2018). Interactions of Surface Exopolysaccharides From *Bifidobacterium* and *Lactobacillus* Within the Intestinal Environment. *Front. Microbiol.* 9:2426. 10.3389/fmicb.2018.02426 30364185 PMC6193118

[B4] Chan Y. G. Kim H. K. Schneewind O. Missiakas D. (2014). The capsular polysaccharide of *Staphylococcus aureus* is attached to peptidoglycan by the LytR-CpsA-Psr (LCP) family of enzymes. *J. Biol. Chem.* 289 15680–15690. 10.1074/jbc.m114.567669 24753256 PMC4140922

[B5] Chen Y. Zhang M. Ren F. (2019). A Role of Exopolysaccharide Produced by *Streptococcus thermophilus* in the Intestinal Inflammation and Mucosal Barrier in Caco-2 Monolayer and Dextran Sulphate Sodium-Induced Experimental Murine Colitis. *Molecules* 24:513. 10.3390/molecules24030513 30708992 PMC6384629

[B6] de Vrese M. Marteau P. R. (2007). Probiotics and Prebiotics: Effects on Diarrhea. *J. Nutr.* 137 803S–811S. 17311979 10.1093/jn/137.3.803S

[B7] Dertli E. Mayer M. J. Narbad A. (2015). Impact of the exopolysaccharide layer on biofilms, adhesion and resistance to stress in *Lactobacillus johnsonii* FI9785. *BMC Microbiol.* 15:8. 10.1186/s12866-015-0347-2 25648083 PMC4326364

[B8] Fukao M. Zendo T. Inoue T. Nakayama J. Suzuki S. Fukaya T. (2019). Plasmid-encoded glycosyltransferase operon is responsible for exopolysaccharide production, cell aggregation, and bile resistance in a probiotic strain, *Lactobacillus brevis* KB290. *J. Biosci. Bioeng.* 128 391–397. 10.1016/j.jbiosc.2019.04.008 31126721

[B9] Han Q. Kong B. Chen Q. Sun F. Zhang H. (2017). In vitro comparison of probiotic properties of lactic acid bacteria isolated from Harbin dry sausages and selected probiotics. *J. Funct. Foods* 32 391–400. 10.1016/j.jff.2017.03.020

[B10] Jiang Y. Yang Z. (2018). A functional and genetic overview of exopolysaccharides produced by *Lactobacillus plantarum*. *J. Funct. Foods* 47 229–240. 10.1016/j.jff.2018.05.060

[B11] Kawai Y. Marles-Wright J. Cleverley R. M. Emmins R. Ishikawa S. Kuwano M. (2011). A widespread family of bacterial cell wall assembly proteins. *EMBO J.* 30 4931–4941. 10.1038/emboj.2011.358 21964069 PMC3243631

[B12] Kim D. H. Kim S. Ahn J. B. Kim J. H. Ma H. W. Seo D. H. (2020). *Lactobacillus plantarum* CBT LP3 ameliorates colitis via modulating T cells in mice. *Int. J. Med. Microbiol.* 310:151391. 10.1016/j.ijmm.2020.151391 32007342

[B13] Le B. Yang S. H. (2018). Efficacy of *Lactobacillus plantarum* in prevention of inflammatory bowel disease. *Toxicol. Rep.* 5 314–317. 10.1016/j.toxrep.2018.02.007 29854599 PMC5977373

[B14] Li F. K. K. Rosell F. I. Gale R. T. Simorre J. P. Brown E. D. Strynadka N. C. J. (2020b). Crystallographic analysis of *Staphylococcus aureus* LcpA, the primary wall teichoic acid ligase. *J. Biol. Chem.* 295 2629–2639. 10.1074/jbc.ra119.011469 31969390 PMC7049971

[B15] Li F. Zhai D. Wu Z. Zhao Y. Qiao D. Zhao X. (2020a). Impairment of the cell wall ligase, LytR-CpsA-Psr protein (LcpC), in methicillin resistant *Staphylococcus aureus* reduces its resistance to antibiotics and infection in a mouse model of sepsis. *Front. Microbiol.* 11:557. 10.3389/fmicb.2020.00557 32425893 PMC7212477

[B16] Li P. Gu Q. Yang L. Yu Y. Wang Y. (2017). Characterization of extracellular vitamin B12 producing *Lactobacillus plantarum* strains and assessment of the probiotic potentials. *Food Chem.* 234 494–501. 10.1016/j.foodchem.2017.05.037 28551266

[B17] Liu J. Gu Z. Zhang H. Zhao J. Chen W. (2019). Preventive effects of *Lactobacillus plantarum* ST-III against *Salmonella* infection. *LWT Food Sci. Tech.* 105:200. 10.1016/j.lwt.2019.02.043

[B18] Lynch K. M. Lucid A. Arendt E. K. Sleator R. D. Lucey B. Coffey A. (2015). Genomics of *Weissella cibaria* with an examination of its metabolic traits. *Microbiology* 161 914–930. 10.1099/mic.0.000053 25678547

[B19] Papadopoulou O. S. Argyri A. A. Varzakis E. E. Tassou C. C. Chorianopoulos N. G. (2018). Greek functional Feta cheese: Enhancing quality and safety using a *Lactobacillus plantarum* strain with probiotic potential. *Food Chem.* 74 21–33. 10.1016/j.fm.2018.02.005 29706334

[B20] Pisano M. B. Viale S. Conti S. Fadda M. E. Deplano M. Melis M. P. (2014). Preliminary Evaluation of Probiotic Properties of *Lactobacillus* Strains Isolated from Sardinian Dairy Products. *BioMed Res. Int.* 2014:286390. 10.1155/2014/286390 PMC4099116 25054135

[B21] Remus D. M. van Kranenburg R. van S. II Taverne N. Bongers R. S. Wels M. (2012). Impact of 4 *Lactobacillus plantarum* capsular polysaccharide clusters on surface glycan composition and host cell signaling. *Microb. Cell Fact.* 11:149. 10.1186/1475-2859-11-149 23170998 PMC3539956

[B22] Spath K. Heinl S. Grabherr R. (2012). Direct cloning in *Lactobacillus plantarum*: Electroporation with non-methylated plasmid DNA enhances transformation efficiency and makes shuttle vectors obsolete. *Microb. Cell Fact.* 11:141. 10.1186/1475-2859-11-141 23098256 PMC3526553

[B23] Terraf M. C. L. Juárez Tomás M. S. Nader-Macías M. E. F. Silva C. (2012). Screening of biofilm formation by beneficial vaginal lactobacilli and influence of culture media components. *J. Appl. Microbiol.* 113 1517–1529. 10.1111/j.1365-2672.2012.05429.x 22897406

[B24] van Baarlen P. Troost F. J. van Hemert S. van der Meer C. de Vos W. M. de Groot P. J. (2009). Differential NF-kappaB pathways induction by *Lactobacillus plantarum* in the duodenum of healthy humans correlating with immune tolerance. *Proc. Natl. Acad. Sci.* 106 2371–2376. 10.1073/pnas.0809919106 19190178 PMC2650163

[B25] Wang L. Fu G. Liu S. Li L. Zhao X. (2019). Effects of oxygen levels and a *Lactobacillus plantarum* strain on mortality and immune response of chickens at high altitude. *Sci. Rep.* 9:16037. 10.1038/s41598-019-52514-w PMC6831595 31690779

[B26] Wang L. Li L. Lv Y. Chen Q. Feng J. Zhao X. (2018). *Lactobacillus plantarum* restores intestinal permeability disrupted by *Salmonella* infection in newly-hatched chicks. *Sci. Rep.* 8:2229. 10.1038/s41598-018-20752-z PMC5797085 29396554

[B27] Wang L. Si W. Xue H. Zhao X. (2017). A fibronectin-binding protein (FbpA) of *Weissella cibaria* inhibits colonization and infection of *Staphylococcus aureus* in mammary glands. *Cell. Microbiol.* 19:e12731. 10.1111/cmi.12731 28125161

[B28] Xiao Y. Liu Y. Chen C. Xie T. Li P. (2020). Effect of *Lactobacillus plantarum* and *Staphylococcus xylosus* on flavour development and bacterial communities in Chinese dry fermented sausages. *Food Res. Int.* 135:109247. 10.1016/j.foodres.2020.109247 32527474

[B29] Yother J. (2011). Capsules of *Streptococcus pneumoniae* and other bacteria: paradigms for polysaccharide biosynthesis and regulation. *Annu. Rev. Microbiol.* 65 563–581. 10.1146/annurev.micro.62.081307.162944 21721938

[B30] Zannini E. Mauch A. Galle S. Gänzle M. Coffey A. Arendt E. K. (2013). Barley malt wort fermentation by exopolysaccharide-forming *Weissella cibaria* MG1 for the production of a novel beverage. *J. Appl. Microbiol.* 115 1379–1387. 10.1111/jam.12329 23957391

[B31] Zhao K. Xie Q. Xu D. Guo Y. Tao X. Wei H. (2018). Antagonistics of *Lactobacillus plantarum* ZDY2013 against *Helicobacter pylori* SS1 and its infection in vitro in human gastric epithelial AGS cells. *J. Biosci. Bioeng.* 126 458–463. 10.1016/j.jbiosc.2018.04.003 29699944

[B32] Zhou X. Qi W. Hong T. Xiong T. Gong D. Xie M. (2018). Exopolysaccharides from *Lactobacillus plantarum* NCU116 Regulate Intestinal Barrier Function via STAT3 Signaling Pathway. *J. Agric. Food Chem.* 66 9719–9727. 10.1021/acs.jafc.8b03340 30134660

[B33] Zhou Y. Cui Y. Qu X. (2019). Exopolysaccharides of lactic acid bacteria: Structure, bioactivity and associations: A review. *Carbohydr. Polym.* 207 317–332. 10.1016/j.carbpol.2018.11.093 30600013

[B34] Živković M. Miljković M. S. Ruas-Madiedo P. Markelić M. B. Veljović K. Tolinački M. (2016). EPS-SJ exopolisaccharide produced by the strain *Lactobacillus paracasei* subsp. paracasei BGSJ2-8 is involved in adhesion to epithelial intestinal cells and decrease on *E. coli* association to Caco-2 cells. *Front. Microbiol.* 7:286. 10.3389/fmicb.2016.00286 27014210 PMC4783416

