# Supplementary figures and images for: Horizontally Acquired Polysaccharide-Synthetic Gene Cluster From Weissella cibaria Boosts the Probiotic Property of Lactiplantibacillus plantarum
Source: Front Microbiol. 2021 Jun 21;12:692957. doi: 10.3389/fmicb.2021.692957 (PMC8256895; doi:10.3389/fmicb.2021.692957)

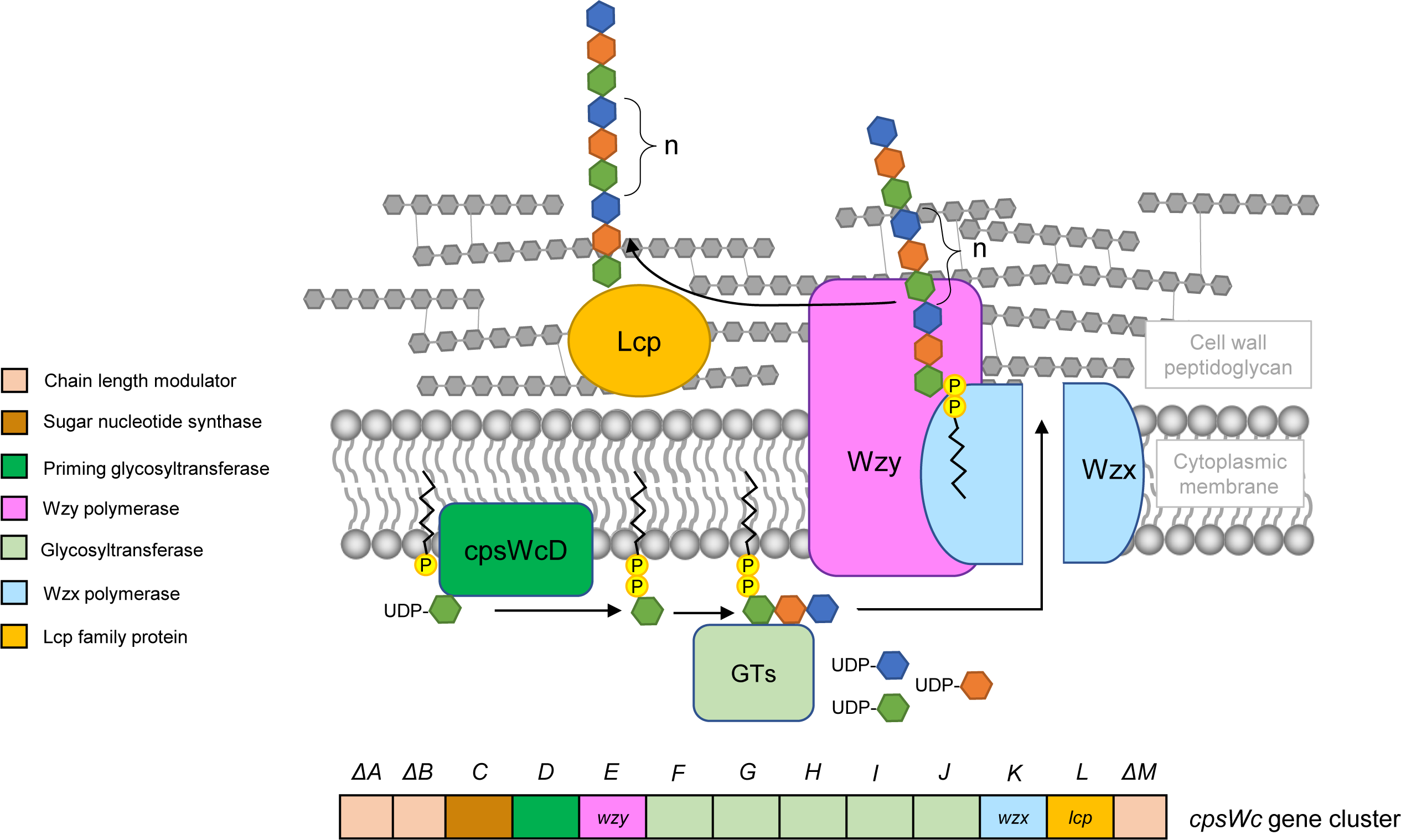

Supplement: Supplementary Figure 1 — The proposed EPS biosynthesis pathway for the cpsWc gene cluster. [file Image_1.tif]

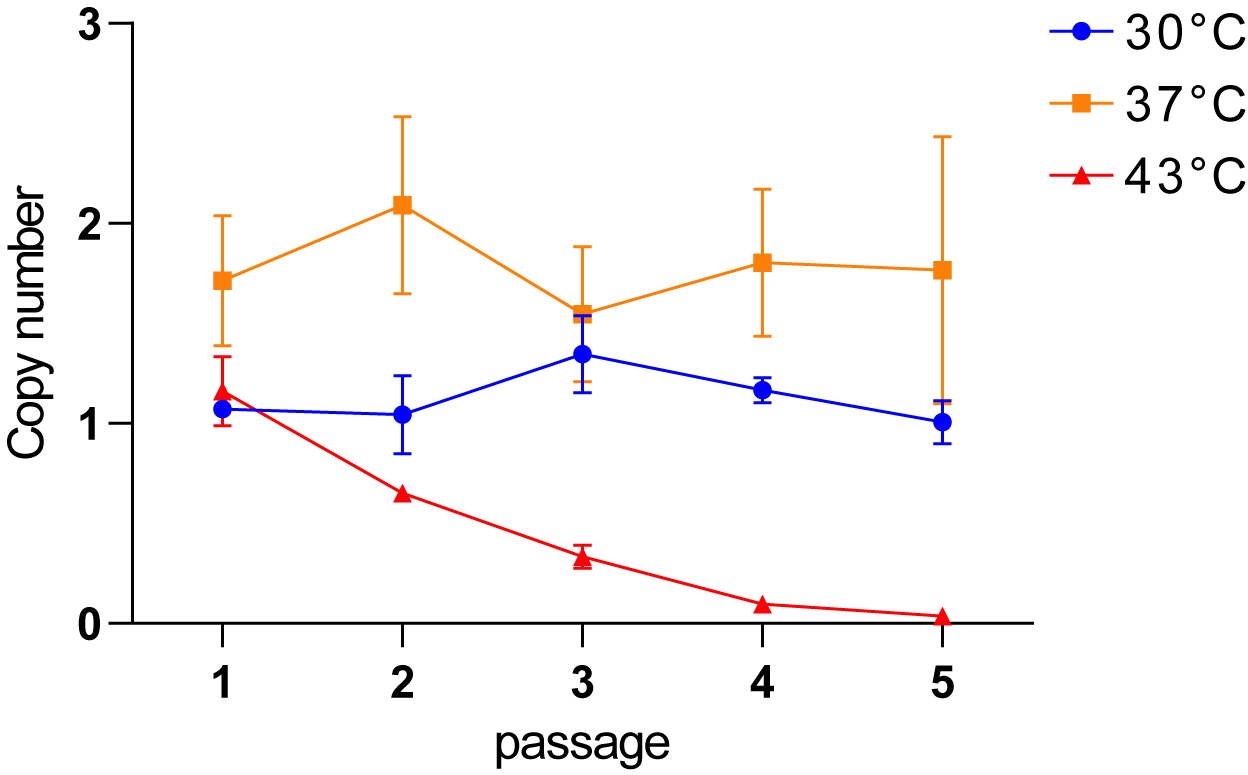

Supplement: Supplementary Figure 2 — Copy number change of pYZ1 during serial passage at different temperatures. The wild-type LTC-113 strain was culture at 30°C, 37°C, and 43°C. The copy number of the plasmid pYZ1 was measured by qPCR. [file Image_2.tif]

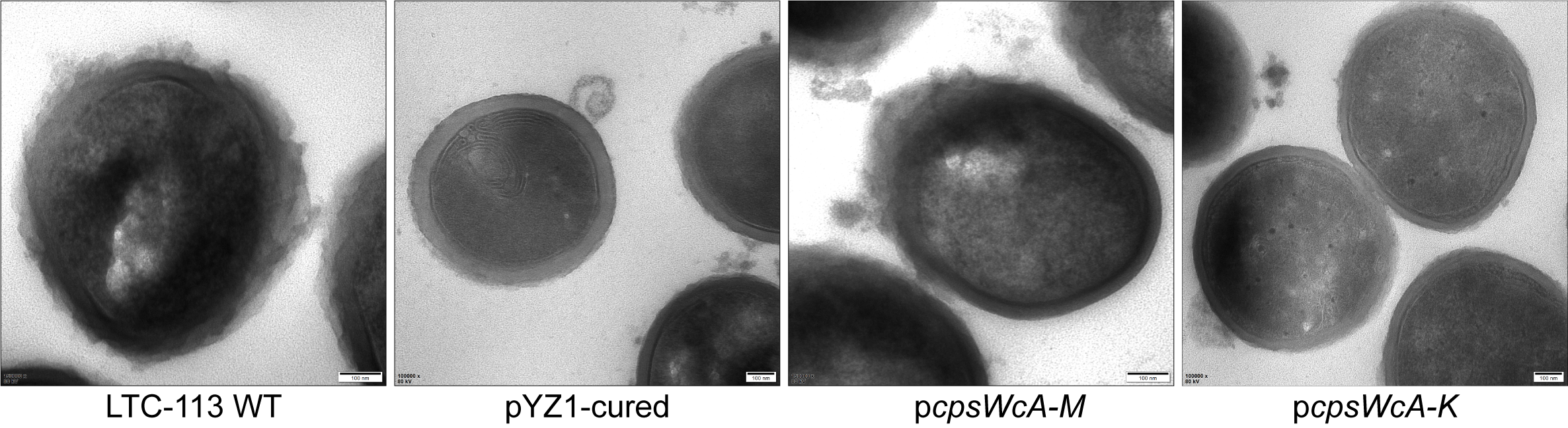

Supplement: Supplementary Figure 3 — Transmission electron microscope analysis of LTC-113 and its derivatives. [file Image_3.tif]

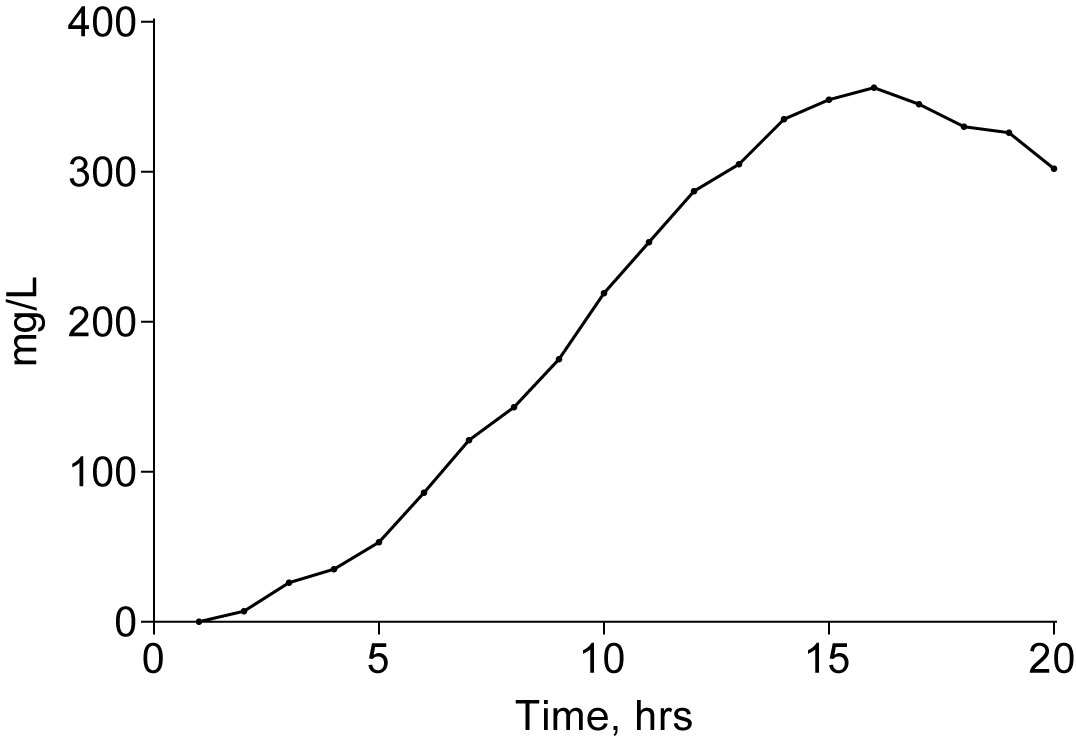

Supplement: Supplementary Figure 4 — The supernatant EPS concentration curve in a 20-hrs time course. [file Image_4.tif]

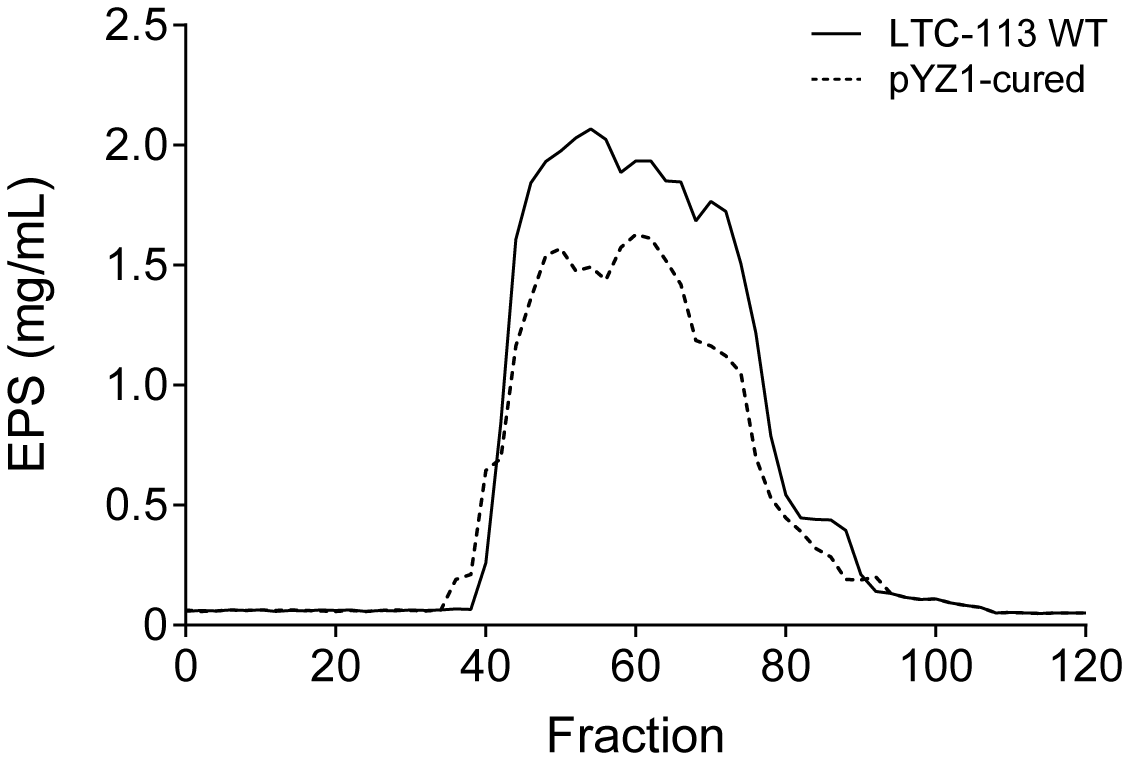

Supplement: Supplementary Figure 5 — Analytical size-exclusion chromatography for EPS from LTC-113 and the pYZ1-cured strain. [file Image_5.tif]

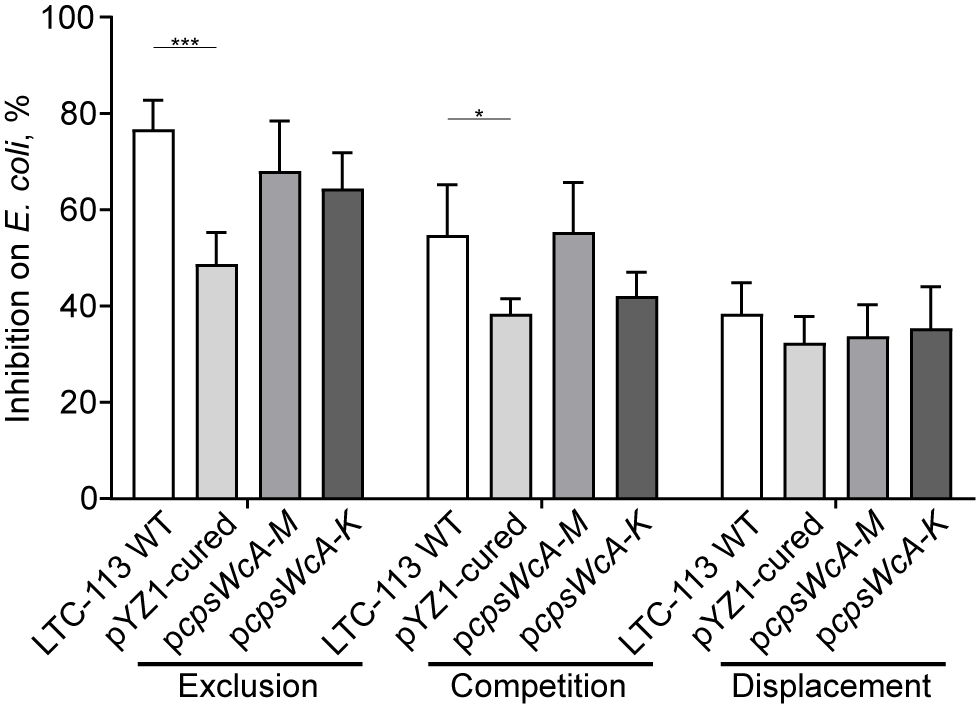

Supplement: Supplementary Figure 6 — Adhesion inhibition of pathogenic E. coli by L. plantarum. The inhibitory activities of LTC-113 and its derivative strains on the adhesion of E. coli in three experimental models, including exclusion, competition, and displacement. Values represent mean ± SD of the mean; *P < 0.05; **P < 0.01; ***P < 0.001. [file Image_6.tif]
