# Supplementary material for: Horizontally Acquired Polysaccharide-Synthetic Gene Cluster From Weissella cibaria Boosts the Probiotic Property of Lactiplantibacillus plantarum
Source: Front Microbiol. 2021 Jun 21;12:692957. doi: 10.3389/fmicb.2021.692957 (PMC8256895; doi:10.3389/fmicb.2021.692957)
Supplement: Supplementary Table 1 — Genomes, plasmids, and oligonucleotides used in this study. [file Table_1.DOCX]

**Table S1. Strains, genomes, plasmids, and oligonucleotides used in this study.**

| ID | Description | Source, sequence, or access link |
| --- | --- | --- |
| Strains | | |
| LTC-113 | Previously identified *L. plantarum* strain with excellent probiotic properties | Laboratory strain (Wang et al., 2018) |
| pYZ1-cured | LTC-113 derivative lost pYZ1 plasmid | This study |
| p*cpsWcA-M* | LTC-113 derivative complemented with a plasmid-borne complete *cpsWc* gene cluster | This study |
| p*cpsWcA-K* | LTC-113 derivative complemented with a plasmid-borne partial *cpsWc* gene cluster | This study |
| Z01 | Previously identified *L. plantarum* strain with low biofilm formation ability | Laboratory strain |
| Z01+ p*cpsWcA-M* | Z01 derivative complemented with a plasmid-borne complete *cpsWc* gene cluster | This study |
| CVCC542 | Pathogenic *S. typhimurium* strain | Laboratory strain |
| O78 | pathogenic *E. coli* strain | Laboratory strain |
| Genomes and plasmids | | |
| LTC-113 | The complete circular genome of *L. plantarum* LTC-113 | https://benchling.com/s/seq-sEmxlqinJNTjtfkQ8CvA |
| pYZ1 | The complete circular sequence of the episomal plasmid pYZ1 | https://benchling.com/s/seq-eFw8aElsy0kWurpPEoAd |
| p*cpsWcA-M* | Complementing plasmid containing the complete *cpsWc* gene cluster | https://benchling.com/s/seq-eFw8aElsy0kWurpPEoAd |
| p*cpsWcA-K* | Complementing plasmid containing the *cpsWc* gene cluster but lack of the *cpsWc*-sepcific LCP family protein | https://benchling.com/s/seq-sRMgr3xrNOflgdxrkVIE |
| Oligonucleotides | | |
| BK-F | Amplification of the *Enterococcus*-originated plasmid backbone | ACTGGCCGTCGTTTTACAACCGGTG |
| BK-R | Amplification of the *Enterococcus*-originated plasmid backbone | GCTCTTGTGCTGTTAGGATATCTTTCTTG |
| AM-F | Amplification of the complete *cpsWc* gene cluster | GATATCCTAACAGCACAAGAGCaggttatgctgacaaatttgtccaaaaattc |
| AM-R | Amplification of the complete *cpsWc* gene cluster | GGTTGTAAAACGACGGCCAGTacgcttgcgtttagttttgtttctc |
| AK-R | Amplification of the *cpsWc* gene cluster but lack of the *cpsWc*-sepcific LCP family protein with AM-F | GGTTGTAAAACGACGGCCAGTtttatcaccaataaaaaggcatcgcac |
| YZ1-RT-F | qPCR quantification of pYZ1 | ctaccgacagacgacatgttcag |
| YZ1-RT-R | qPCR quantification of pYZ1 | ctgcagacaaaggccaagtttgc |
| gyrB-RT-F | qPCR quantification of *gyrB* | gtacgacgagtagccaaggactc |
| gyrB-RT-R | qPCR quantification of *gyrB* | cccgttttttcttgaatatcaacagg |
| GAPDH-RT-F | qPCR quantification of GAPDH | CTCCTCCTGTTCGACAGTCA |
| GAPDH-RT-R | qPCR quantification of GAPDH | CGACCAAATCCGTTGACTCC |
| IL-8-RT-F | qPCR quantification of IL-8 | TGGCTCTCTTGGCAGCCTTC |
| IL-8-RT-R | qPCR quantification of IL-8 | TGCACCCAGTTTTCCTTGGG |
